# Indoxyl sulfate exacerbates chronic inflammation and susceptibility to severe infection via modulating T-cell CD73/adenosine signaling in chronic kidney disease

**DOI:** 10.1080/0886022X.2026.2706213

**Published:** 2026-08-05

**Authors:** JiLai Cao, Yuxin Nie, Xuesen Cao, Xiaohong Chen, Jin Bo, Yaqiong Wang, Jianzhou Zou, Xiaoqiang Ding, Bo Shen, Fangfang Xiang

**Affiliations:** ^a^ School of Clinical Medicine, Shanghai Medical College, Fudan University, Shanghai, China; ^b^ Department of Nephrology, Zhongshan Hospital, Fudan University, Shanghai, China; ^c^ Shanghai Key Laboratory of Renal Disease and Blood Purification, Shanghai, China; ^d^ Shanghai Medical Center of Kidney, Shanghai, China; ^e^ Shanghai Institute of Kidney and Dialysis, Shanghai, China

**Keywords:** Chronic kidney disease, indoxyl sulfate, CD73, adenosine, T cell dysfunction

## Abstract

**Background:**

Chronic kidney disease (CKD) is a major global health issue. Cardiovascular events and infections drive mortality in end-stage renal disease via immune dysregulation. The role of T-cell purinergic signaling and its modulation by indoxyl sulfate (IS) in CKD remains unclear.

**Methods:**

Male Sprague-Dawley rats underwent 5/6 nephrectomy. The CKD + IS group received daily IS (100 μg/kg, intraperitoneally) for 24 weeks; control group received saline. Samples were collected 2–6 h post-injection. Sepsis was induced via cecal ligation and puncture. Splenic T-cell mRNA cluster of differentiation (CD) 73, CD39, A2A receptor (A2AR), P2X purinergic receptor (P2RX7), nuclear factor kappa-B (NF-κB) was quantified by quantitative real-time polymerase chain reaction. Plasma cytokines, vascular injury markers (asymmetric dimethylarginine (ADMA), intercellular adhesion molecule 1 (ICAM-1)), and myocardial markers (cardiac troponin T (cTnT), B-type natriuretic peptide (BNP)) were measured by enzyme-linked immunosorbent assay. Peripheral purine metabolites (adenosine triphosphate, adenosine diphosphate, adenosine monophosphate, adenosine (ADO)) were analyzed via ultra-high performance liquid chromatography-tandem mass spectrometry. Survival was monitored for 72 h.

**Results:**

IS exacerbated baseline microinflammation, elevating plasma cytokines (interleukin 2 (IL-2), IL-6, IL-10, IL-17, tumor necrosis factor-alpha (TNF-α)) and T-cell NF-κB. This coincided with disrupted anti-inflammatory purinergic signaling, characterized by downregulated CD73/A2AR and reduced ADO. Post-infection, the CKD + IS group showed profound CD73 suppression and ADO depletion. Despite blunted cytokine surges, this group exhibited aggravated vascular and myocardial injury (elevated ADMA, ICAM-1, cTnT, BNP) and significantly higher 72-hour mortality.

**Conclusions:**

Indoxyl sulfate disrupts the T-cell CD73/ADO axis, promoting basal microinflammation while impairing anti-infective immunity and exacerbating organ damage during sepsis. Targeting the IS–CD73–ADO pathway offers a therapeutic strategy for restoring immune homeostasis in uremia.

## Introduction

Chronic kidney disease (CKD) has emerged as a formidable global health challenge. Recent epidemiological data indicate that as of 2023, approximately, 788 million individuals over the age of 20 are affected by CKD, with an age-standardized prevalence of 14.2% – representing a substantial increase of roughly 3.5% since 1990 [1]. As the disease advances, patients experience an irreversible decline in renal function, ultimately leading to end-stage renal disease (ESRD) [2]. Although renal replacement therapy can prolong survival, conventional dialysis fails to completely clear metabolic waste products, leading to a clinical state known as ‘residual syndrome’ [3]. Previous studies have identified cardiovascular and cerebrovascular diseases (CVDs), along with infections, as the primary causes of mortality among CKD patients [4,5]. Central to this pathological progression is the systemic accumulation of uremic toxins, uremic toxins can be categorized into four main groups: small molecule, medium molecule, large molecule and protein-binding toxins (PBUTs), the last group constitutes approximately 25% of all identified uremic toxins. Due to their strong affinity for plasma proteins, PBUTs are notoriously resistant to clearance by conventional dialysis. Indoxyl sulfate (IS), a prototypical PBUT, binds extensively to circulating albumin [6], severely limiting its dialytic removal. As renal function deteriorates, serum IS concentrations progressively accumulate, thereby exacerbating CKD progression and contributing to adverse clinical outcomes [7].

Compared to healthy individuals, patients with renal insufficiency exhibit pronounced immune disorders. The immune dysfunction observed in CKD patients is independent of underlying comorbidities and manifests early in the course of renal insufficiency [8]. Both the innate and adaptive immune systems are compromised in this population [9]. Impairment of the innate immune system primarily presents as dysfunction of various pattern recognition receptors, alongside diminished antigen-presenting capacities of dendritic cells and macrophages. T cells, which are crucial components of the adaptive immune system, experience dysfunction that significantly affects acquired immunity. This defect is evidenced by significant alterations in T-cell subset distributions, including a decreased CD4+/CD8+ ratio and a skewed Th1/Th2 balance, as well as aberrant cytokine production profiles. Such an immunological imbalance disrupts critical signaling cascades within immune pathways. Furthermore, there is an accelerated depletion of naive and memory T cells; the magnitude of this reduction correlates directly with the severity of azotemia, oxidative stress, and systemic inflammation during CKD progression [10].

The purinergic signaling pathway serves as a critical regulator of immune cell function. T cells co-express P1 receptors, which are primarily activated by adenosine (ADO) to mediate anti-inflammatory responses, and P2 receptors, which are responsive to extracellular adenosine triphosphate (ATP)/adenosine diphosphate (ADP) and transduce pro-inflammatory signals [11]. Research indicates that during the acute phase of tissue injury, a substantial release of ATP from the damaged tissue [12,13] leads to the activation of P2-type receptors in significant quantities, which exhibit an pro-inflammatory response. The cluster of differentiation (CD)39/CD73–ADO axis constitutes a pivotal purinergic checkpoint in T cells, wherein CD39 hydrolyzes extracellular ATP to adenosine monophosphate (AMP), followed by the conversion of AMP to ADO by CD73. The resulting ADO engages A2A receptor (A2A) receptors to dampen excessive T-cell activation, thereby preserving immune homeostasis [14,15]. Building on this framework, our prior clinical investigation revealed a significant downregulation of CD73 expression on T cells in hemodialysis patients, an alteration independently associated with chronic inflammation, cardiovascular events, and susceptibility to infection [16]. These findings suggest that impaired CD73/ADO signaling may play an important role in CKD. However, it remains unclear how T-cell purinergic signaling dynamically evolves during CKD progression, and whether the uremic toxin IS directly modulates the CD73/ADO axis. Elucidating these mechanisms is critical for deciphering the pathophysiology of CKD-associated immune dysfunction. Accordingly, the present study aimed to determine whether IS disrupts T-cell CD73/ADO signaling, thereby driving chronic inflammation and exacerbating susceptibility to infection in a uremic rat model.

## Materials and methods

*Experimental animals*: Multiple male Sprague-Dawley (SD) rats, specific pathogen-free-grade, weighing between 200 and 220 g, were selected for the study. All rats were randomly assigned to control group, CKD group, and CKD + IS group, with 15 rats in each group, five rats were used for experiments without cecal ligation and puncture (CLP) induction, five rats were euthanized and blood and tissue samples were collected, another five rats were used to observe the 72-h mortality rate. Following a one-week period of adaptive feeding, the rats were utilized in the experiments. Upon completion of the experiment, rats were deeply anesthetized with isoflurane (5% for induction, 2% for maintenance) until loss of consciousness and absence of pedal withdrawal reflex. Subsequently, euthanasia was performed by cervical dislocation. All animal procedures received approval from the Ethics Committee and Ethics Review Committee for Animal Experimentation of Zhongshan hospital and adhered strictly to established ethical standards for animal research.

### Construction of CKD rat model

CKD models were established in the CKD and CKD + IS group via 5/6 nephrectomy. After isoflurane anesthesia, the lower and upper thirds of the left kidneys were resected in week 0, and the right kidney was removed in week 12. The second surgery was postponed due to the lockdown policy during the severe acute respiratory syndrome Coronavirus 2 pandemic. The control group underwent sham operations at the same intervals, with only renal capsule dissection and no parenchymal resection. Following the successful establishment of the 5/6 Nx model, rats in the IS group received daily intraperitoneal injections of IS (100 μg/kg) for 24 consecutive weeks, while the CKD control group was administered an equivalent volume of normal saline daily. Biological samples were collected within 2–6 h after the final injection. Details about the CKD model are provided in the Supplementary Materials.

### Construction of cecal ligation and puncture infection model

Rats under 1% pentobarbital sodium anesthesia were secured, and a left lower abdominal incision was made to enter the peritoneal cavity. The cecum was exteriorized, punctured with a sterile needle, and gently squeezed to expel a small amount of feces before being returned to the abdomen. The incision was sutured in layers, followed by subcutaneous injection of 1 mL pre-warmed saline and buprenorphine for analgesia. The control group underwent identical laparotomy without ligation or puncture. Twenty-four hours after the CLP procedure, five rats of each group were euthanized and blood and tissue samples were collected. Another five rats in each group were used to observe the 72-h mortality rate. Details about the CLP model are provided in the Supplementary Materials.

### Detection indicators and methods

#### Plasma index detection

Peripheral blood inflammatory factors (IL-2, IL-6, IL-10, IL-17, TNF-α), myocardial injury markers (cardiac troponin T (cTnT), B-type natriuretic peptide (BNP)), and vascular injury markers (intercellular adhesion molecule 1 (ICAM-1), asymmetric dimethylarginine (ADMA)) were detected by enzyme-linked immunosorbent assay method, and peripheral blood ATP, ADP, AMP, and ADO concentrations were detected by ultra-high performance liquid chromatography-tandem mass spectrometry method. Details are provided in the Supplementary Materials.

#### Tissue molecular detection

T cells from the spleen of rats were isolated, and the mRNA expression levels of purinergic signal-related molecules such as CD73, A2AR, CD39, and P2X purinergic receptor (P2RX7) were detected by quantitative real-time polymerase chain reaction. Details are provided in the Supplementary Materials.

### Statistical analysis

Data analysis was performed using SPSS software (version 26.0, Armonk, NY) and GraphPad Prism (version 9.0, GraphPad Software, San Diego, CA). Continuous variables are presented as mean ± standard deviation (S.D.). Normality of the data was assessed using the Shapiro–Wilk test, and homogeneity of variance was evaluated using Levene’s test. For multi-group comparisons, one-way analysis of variance (ANOVA) was performed, followed by Tukey’s post hoc test for pairwise comparisons to strictly control the family-wise error rate. To evaluate the magnitude and precision of the differences, effect sizes were calculated as Cohen’s *d* along with their 95% confidence intervals (95% CIs). Animal survival curves were analyzed using the Kaplan–Meier method with the log-rank test. A two-tailed *p* < 0.05 was considered statistically significant.

## Results

### IS aggravates the basal microinflammation and severe postoperative infection after CLP in CKD rats

**Figure 1.**
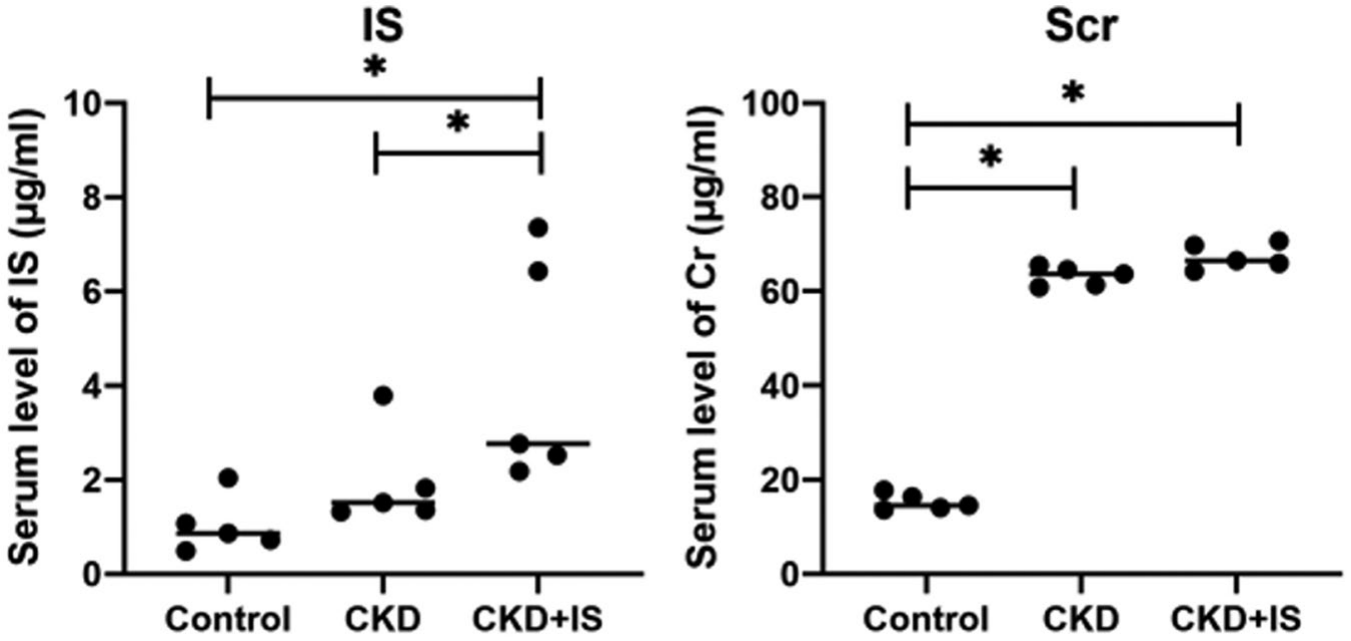
The levels of IS and serum creatinine in peripheral blood in different groups. After 24 weeks of nephrectomy (the control group underwent a sham operation), rats in CKD + IS group were intraperitoneally injected with 100 μg/kg of IS. Serum levels of both of IS and creatinine increased (*n* = 5 per group) (*p < 0.05).

**Figure 2.**
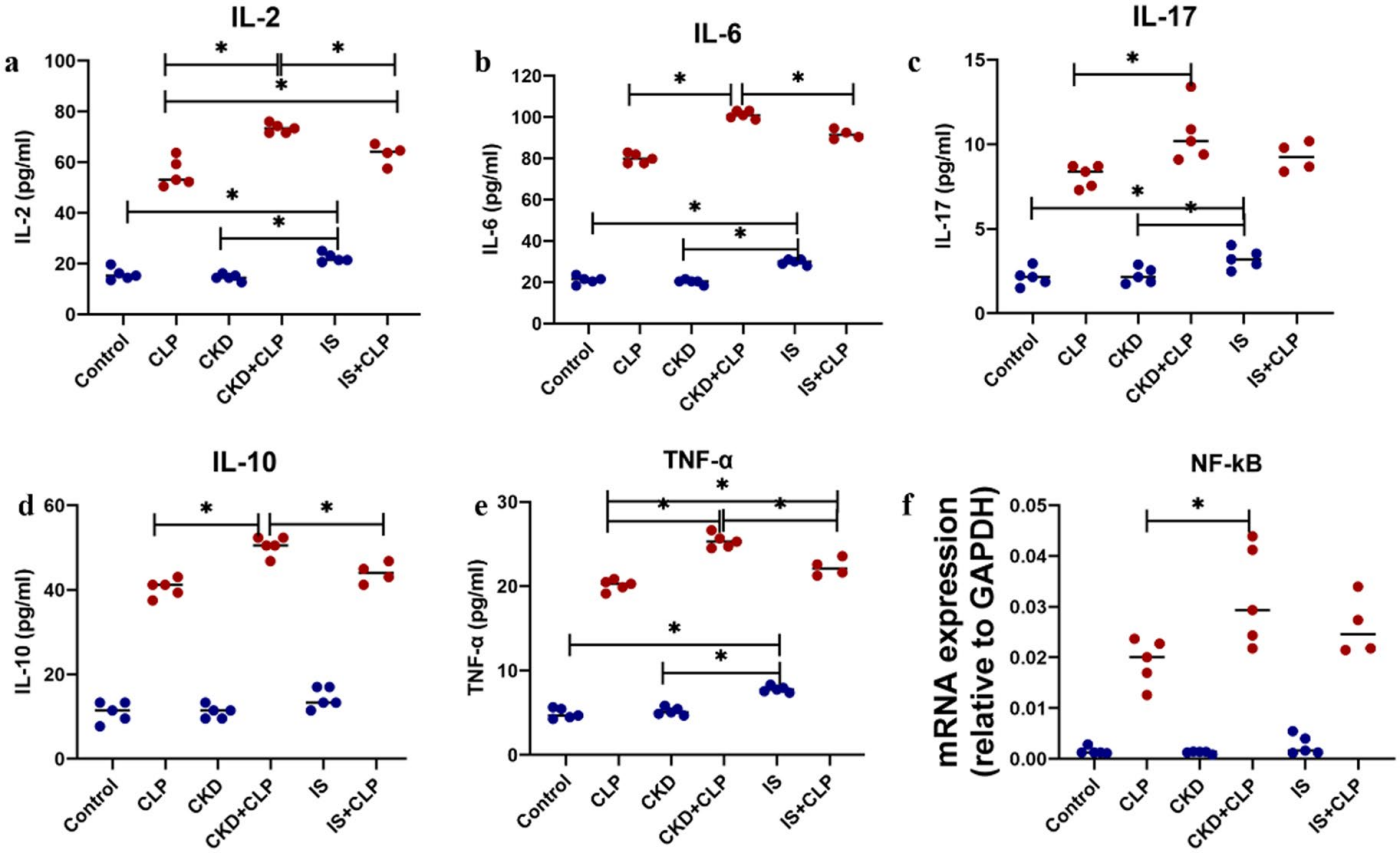
The levels of inflammatory factors in peripheral blood in CKD + IS group. Peripheral blood inflammatory factors were detected by enzyme-linked immunosorbent assay method. The levels of inflammatory factors were upregulated in the CKD group, with further elevation in the CKD + IS group. After CLP surgery, the levels of inflammatory factors increased in the CKD and CKD + IS group, while the magnitude of increase in the CKD + IS group was slightly smaller (*p < 0.05).

Compared with the control group, the levels of serum creatinine in the CKD group and the CKD + IS group were elevated significantly (*p* < 0.05). While IS levels showed only a marginal increase in the CKD group and cytokine levels remained largely unaltered, the CKD + IS group exhibited a significant elevation in IS compared to the control group (control vs. CKD + IS: Cohen’s *d* = 1.61, 95% CI: [0.33, 3.00], *p* = 0.018). Furthermore, circulating inflammatory mediators were significantly upregulated in the CKD + IS group relative to the control group specifically manifested in IL-2 (Cohen’s *d* = 3.20, 95% CI: [1.20, 5.20], *p* < 0.001), IL-6 (Cohen’s *d* = 5.19, 95% CI: [2.36, 8.01], *p* < 0.001), IL-17 (Cohen’s *d* = 1.93, 95% CI: [0.55, 3.44], *p* = 0.019), and TNF-α (Cohen’s *d* = 5.78, 95% CI: [3.91, 8.12], *p* < 0.001). Nuclear factor kappa-B (NF-κB) levels showed an upward trend, but this did not reach statistical significance (Figures 1 and 2).

**Figure 3.**
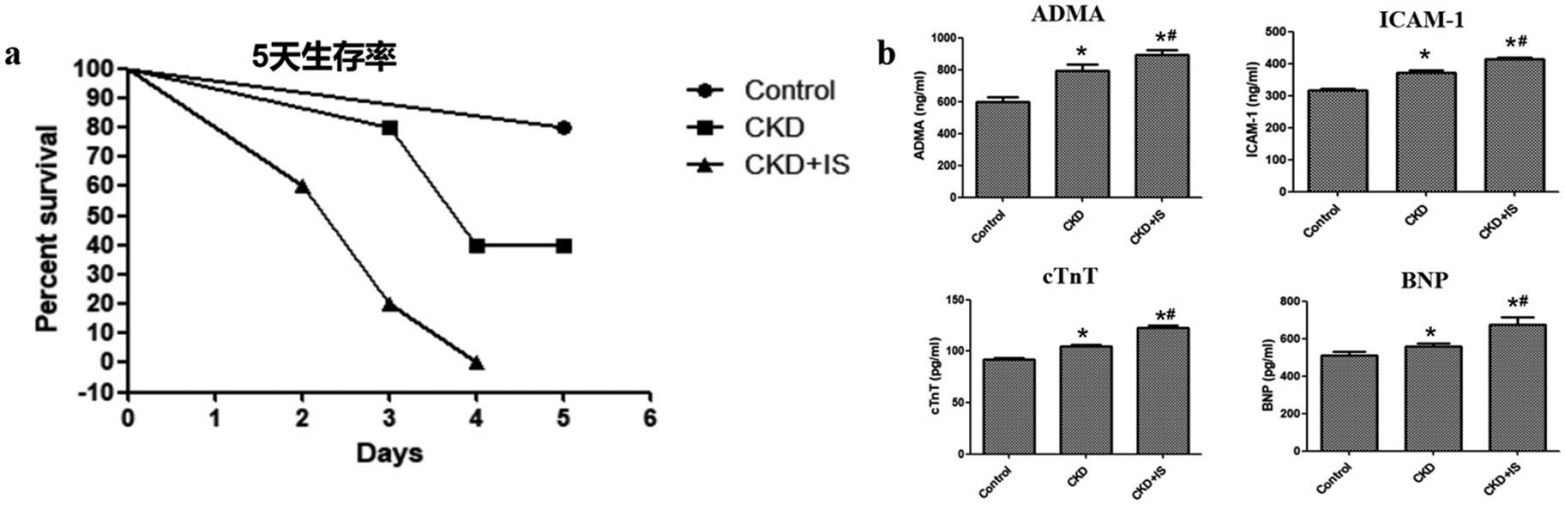
Survival curves and assessment of tissue injury markers. (a) The mortality rate within 72 h of CKD group showed slight increase, while CKD + IS group increased significantly. (b) Vascular and myocardial injury markers increased in both groups, IS + CKD group showed a greater increase (*p < 0.05, compared to the control group; *^,#^*p* < 0.05, compared to the CKD group).

Following CLP surgery, cytokine levels rose across all groups relative to their baseline. Compared with the control group, both of the CKD group and the CKD + IS group showed substantially higher levels of inflammatory factors. However, when compared to the CKD group, the CKD + IS group demonstrated a blunted incremental surge in pro-inflammatory cytokines (IL-2: Cohen’s *d* = 3.34, 95% CI: [1.37, 5.15], *p* = 0.01; IL-6: Cohen’s *d* = 4.51, 95% CI: [1.96, 6.69], *p* < 0.001; TNF-α: Cohen’s *d* = 3.33, 95% CI: [1.33 5.27], *p* < 0.001) (Figure 2). Furthermore, post-CLP 72-h mortality rates were markedly higher in both CKD and CKD + IS group. Additionally, markers of vascular injury (ADMA, ICAM-1) and myocardial injury (cTnT, BNP) were significantly exacerbated in the CKD + IS group (CKD vs. CKD + IS: ADMA: *p* = 0.007; ICAM-1: *p* = 0.001; cTnT: *p* < 0.001; BNP: *p* < 0.001) (Figure 3).

### IS exacerbates the abnormal CD73/ADO signaling during the CKD process

**Figure 4.**
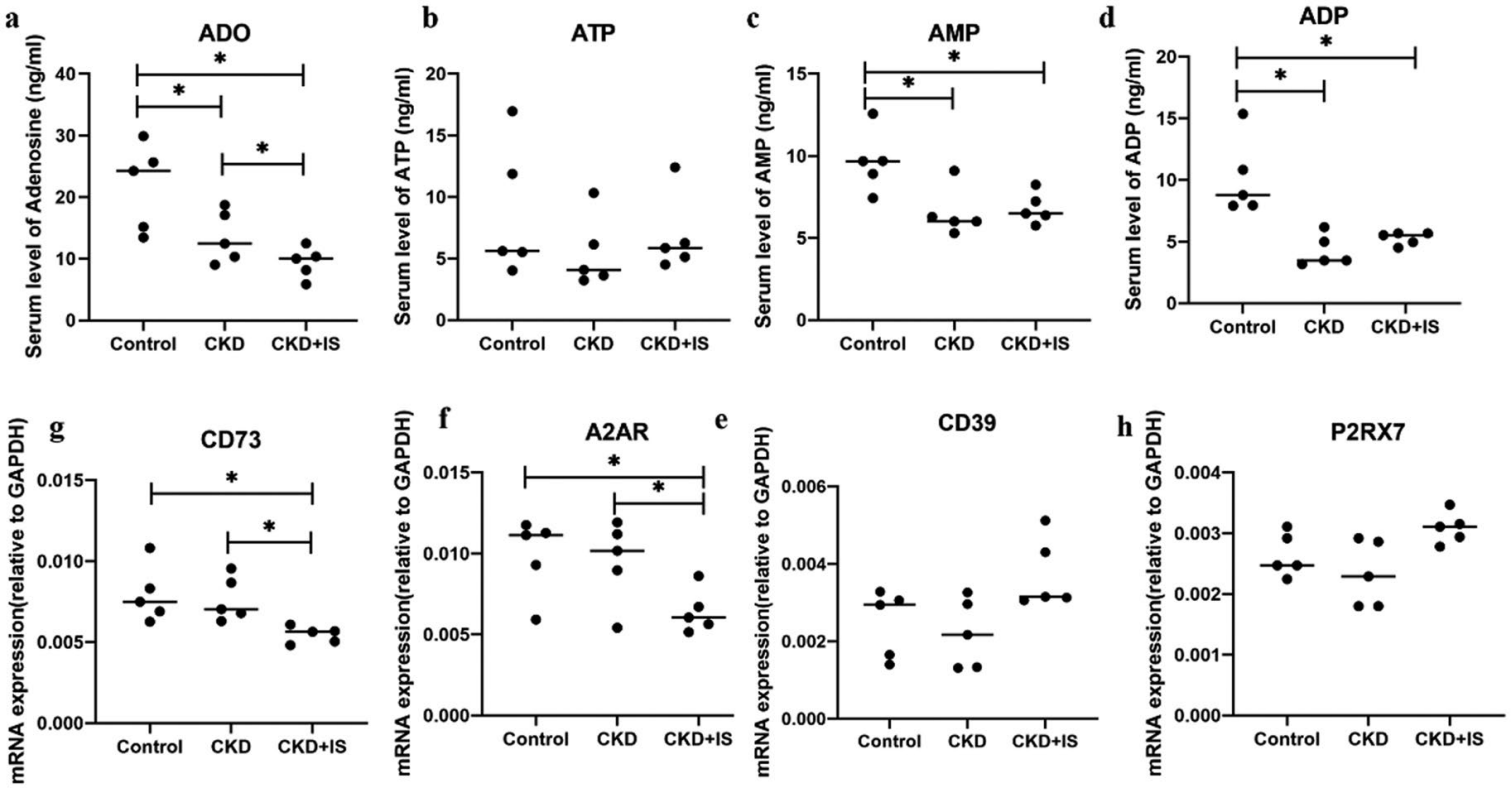
CD73-ADO signaling was aggravated by IS. (a) ADO decreased in CKD and CKD + IS group, CKD + IS group showed a greater decrease than CKD group. (b) There was no significant differences in ATP levels among the three groups. (c, d) Both ADP and AMP decreased in CKD and CKD + IS group compared with control group. (e–h) In CKD group, mRNA transcription levels of CD39, CD73, A2AR, and P2RX7 showed no statistically significant difference compared with control group. In CKD + IS group, mRNA transcription levels of CD39 and P2RX7 showed an upward trend, compared with control group, while CD73 and A2AR decreased significantly (*p* < 0.05).

Significant reductions in CD73/ADO signaling-associated purine nucleotide metabolites were observed in both the CKD and CKD + IS group when benchmarked against the control group, which can be reflected by changes in levels of ADO (CKD + IS vs. control: Cohen’s *d* = 2.32, 95% CI: [0.58, 4.52], *p* = 0.005), ADP (CKD + IS vs. control: Cohen’s *d* = 2.17, 95% CI: [0.34, 4.32], *p* = 0.005), and AMP (CKD vs. control: Cohen’s *d* = 1.85, 95% CI: [0.31, 3.38], *p* = 0.016), while ATP level showed no significant changes. Compared with the control group, there were no significant changes in splenic T-cell mRNA expression of key purinergic cascade components, such as CD73, CD39, A2AR, and P2RX7 in the CKD group. However, significant changes occurred in the CKD + IS group, CD73 (CKD vs. CKD + IS: Cohen’s *d* = 2.14, 95% CI: [0.58, 3.51], *p* < 0.05) and A2AR (CKD vs. CKD + IS: Cohen’s *d* = 1.52, 95% CI: [0.04, 2.72], *p* < 0.05) were significantly downregulated, while the pro-inflammatory ectonucleotidase CD39 and receptor P2RX7 showed an upward transcriptional trend (Figure 4).

### The abnormal CD73/ADO signaling in CKD rats intervened by IS further aggravated after CLP

**Figure 5.**
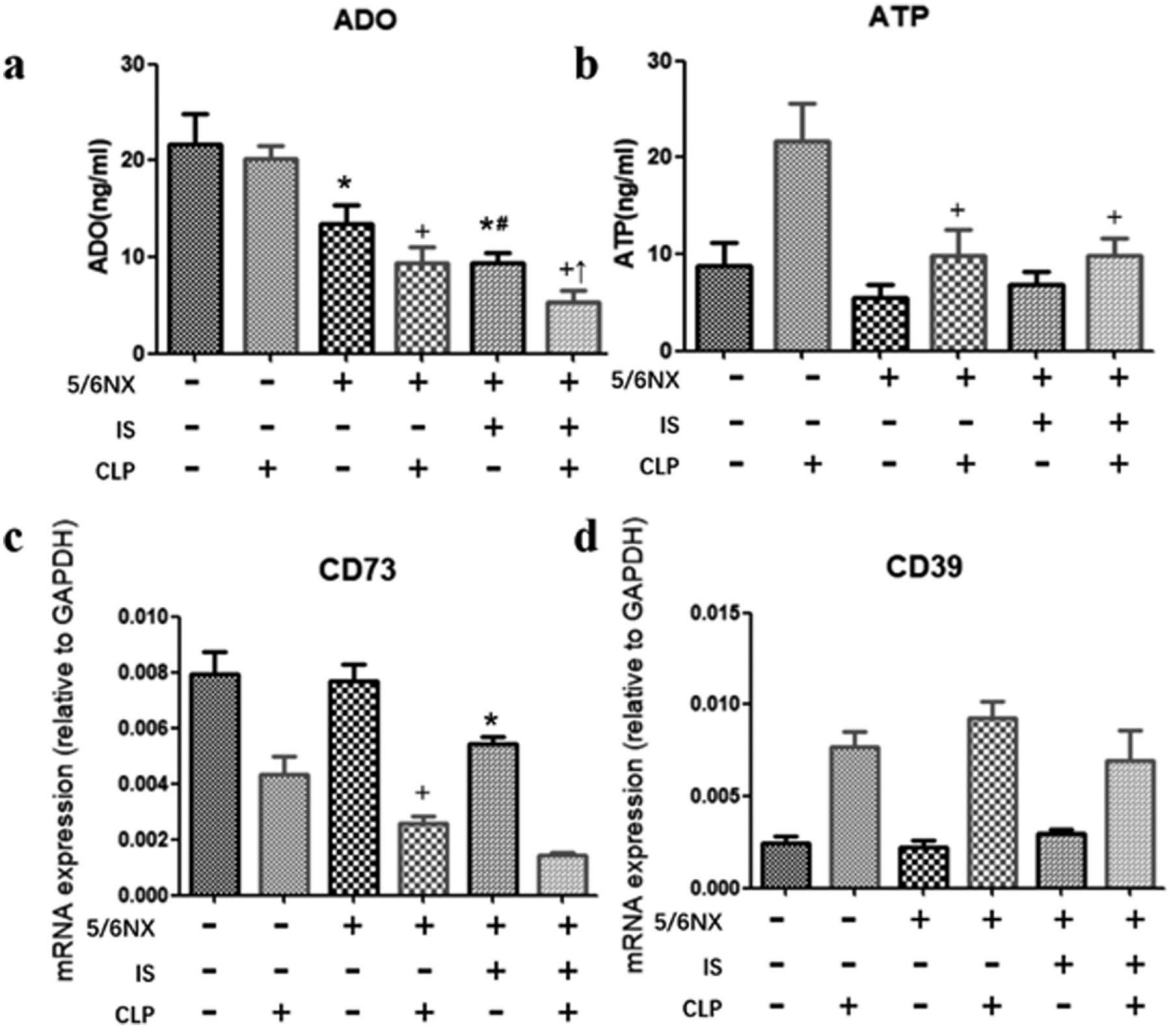
CD73-ADO signaling was further aggravated after CLP surgery. (a) ADO decreased after CLP surgery, the CKD and CKD + IS group showed significant difference and the CKD + IS group decreased more markedly. (b) The ATP increased after CLP surgery in all the three groups, while the magnitude in CKD and CKD + IS group was significantly smaller than that of the control group. (c) mRNA transcription levels of CD73 decreased after CLP surgery, CKD group and CKD + IS group showed significant difference. (d) All the groups showed obvious increase in mRNA transcription levels of CD39 after CLP surgery, while no significant difference was observed among groups. *p < 0.05, compared to the control group; *^,#^*p* < 0.05, compared to CKD group; ^+^*p* < 0.05, compared to the control group after CLP surgery; ^+,↑^*p* < 0.05, compared to CKD group after CLP surgery.

After CLP surgery, extracellular ATP concentrations in the peripheral blood increased across all cohorts compared to baseline levels; however, this compensatory elevation was significantly blunted in rats with a CKD background relative to non-CKD controls (Cohen’s *d* = 1.57, 95% CI: [0.08, 2.98], *p* < 0.05). Conversely, circulating ADO levels declined globally post-CLP, with CKD rats exhibiting a more profound depletion than their non-CKD counterparts (Cohen’s *d* = 3.34, 95% CI: [1.27, 5.33], *p* < 0.001). At the transcriptional level, splenic T-cell CD39 mRNA expression was uniformly upregulated post-infection, showing no significant divergence between the CKD and non-CKD group. In contrast, CD73 transcription was markedly downregulated post-CLP, with the most severe suppression observed in the CKD background (control vs. CKD: Cohen’s *d* = 1.74, 95% CI: [0.21, 3.20], *p* = 0.021). Notably, exogenous IS administration further exacerbated this signaling impairment under septic conditions. Compared with the untreated CKD group, the CKD + IS group exhibited a decreasing trend in splenic T-cell CD73 transcription and systemic ADO levels (Figure 5).

## Discussion

This study elucidates the critical impact of the uremic toxin IS on T-cell purinergic signaling. By utilizing a classic 5/6 nephrectomy model, we provide the first transcriptional evidence that IS disrupts this pathway by modulating key components of the CD39/CD73–ADO axis. This disruption mediates the micro-inflammatory state and subsequently inhibits the inflammatory response following infection, thereby exacerbating tissue damage and increasing susceptibility to severe infections. Collectively, these findings underscore the critical role of uremic toxins in dysregulating purinergic signaling. Furthermore, they highlight the immunological deficits associated with uremia and offer a novel molecular framework for understanding the synergistic mechanisms underlying concurrent infection and cardiovascular injury in patients with CKD.

Notably, although circulating IS levels were modestly elevated in CKD group compared to controls, this did not translate into a corresponding increase in systemic cytokine production. Conversely, in the absence of infection, exogenous IS administration profoundly disrupted the purinergic signaling balance. Specifically, we observed a significant downregulation in the mRNA expression of CD73 and the P1-type receptor A2AR – key components of the anti-inflammatory arm – accompanied by a concurrent reduction in plasma ADO levels. In contrast to the baseline CKD state, exogenous IS treatment upregulated pro-inflammatory mediators, as evidenced by increased extracellular ATP and AMP levels, alongside elevated mRNA expression of CD39 and the P2-type receptor P2RX7. Concurrently, we observed significant elevations in both pro-inflammatory cytokines (IL-2, IL-6, IL-17, and TNF-α) and the anti-inflammatory cytokine IL-10. Furthermore, NF-κB mRNA expression in splenic T cells showed an increasing trend. These findings suggest that IS can disrupt the immune homeostasis and exacerbate the micro-inflammatory state in CKD rats, aligning with previous literature. For instance, a cross-sectional study by Olivier et al. demonstrated a positive correlation between plasma IS concentrations and microinflammation in non-diabetic patients with CKD [17]. It should be noted that the spleen is a highly perfused secondary lymphoid organ and a major reservoir for recirculating T cells. Accordingly, transcriptional changes of molecules such as CD73 and CD39 in splenic T cells can, to a certain extent, reflect the state of systemic immune activation in uremia. However, mRNA expression is not equivalent to surface protein abundance or enzymatic activity. Moreover, multiple layers of buffering and regulation exist between local nucleotide metabolism in the spleen and circulating purine metabolite concentrations in the peripheral blood. Therefore, gene expression in splenic T cells does not directly dictate the levels of metabolites in the peripheral circulation.

Prior studies show that IS upregulates pro-inflammatory cytokines, inhibits immune checkpoints, induces pro-inflammatory T-cell phenotypes, activates aryl hydrocarbon receptor (AhR), and causes DNA damage, thereby worsening micro-inflammation [18]. IS also induces nephrotoxicity via reactive oxygen species (ROS) generation [19], antioxidant depletion [20], fibrosis, and inflammation. In human kidney-2 cells, IS activates NF-κB to inhibit proliferation, induces p53-mediated senescence, and promotes fibrosis via transforming growth factor-beta 1 and plasminogen activator inhibitor-1 [19,21]. Gut-related studies show IS disrupts intestinal homeostasis and induces epithelial inflammation, raising TNF-α and IL-6 [22].

CD39/CD73 is a critical metabolic enzyme for T-cell purinergic signaling. By regulating the balance between extracellular ATP and ADO, it guides the transformation of immune regulation toward pro-inflammatory or anti-inflammatory signals, serving as the core signal for maintaining immune homeostasis [23–25]. Studies have shown that dysregulation of the CD73–ADO plays a key role in critical physiological processes such as ischemic preconditioning, leukocyte migration, osteogenesis, and inflammation [26,27], and is related to the occurrence and development of systemic diseases such as pancreatic ductal adenocarcinoma [28], glaucoma [29], and hematological malignancies [30]. Our previous research has shown that the expression of CD73 on T cells is decreased in hemodialysis patients, which is associated with CVD, infection and death. In this study, we confirmed in the animal model that CKD is accompanied by aberrant CD73–ADO signaling, and IS further exacerbates this dysfunction. Notably, IS exposure impaired host defense against infection and exacerbated organ damage in CKD rats, a phenomenon closely linked to CD73–ADO signaling dysregulation. Specifically, following CLP-induced sepsis, ATP levels were significantly lower in CKD rats compared to controls. Furthermore, IS intervention aggravated this compromised immune response post-infection, as evidenced by reduced cytokine production and a further decline in CD73–ADO signaling. Meanwhile, vascular injury markers and myocardial injury markers in CKD rats significantly increased after IS intervention. These results suggest that IS may disrupt the CD73–ADO signaling cascade to simultaneously impair anti-infective immunity and exacerbate tissue damage, thereby leading to adverse clinical outcomes. This pathophysiological process aligns closely with the high incidence of severe infections and elevated cardiovascular mortality observed in patients with ESRD.

The results indicate that in the progression of CKD, IS aggravates the dysregulation of the purinergic signaling pathway in T cells, mainly manifested as the decreased expression of CD73 and the reduced generation of ADO. Additionally, after CLP, IS further aggravates the abnormal CD73–ADO signaling in CKD rats, which may be related to immunosuppression and organ damage after infection. Previous studies have shown that CD39 and CD73 are widely expressed across various tissues [31]. By sequentially degrading ATP, ADP, and AMP into ADO, they regulate purinergic receptor activation and serve as a crucial ‘immune switch’ that transitions the microenvironment from pro-inflammatory to anti-inflammatory states [32], thereby participating in the pathogenesis of multiple systemic disease [33]. For instance, Francois et al. reported that while CD4+ T lymphocytes in the intestines of uninfected rats express CD39 and CD73, acute infection of *Toxoplasma gondii* downregulates CD73 expression. This reduction impairs ADO production and intestinal barrier function, ultimately intensifying the inflammatory response [34]. Similarly, wild-type rats pretreated with specific CD73 inhibitors prior to renal ischemia exhibit exacerbated inflammation, aggravated organ damage, and increased mortality [35]. Consequently, targeting ADO metabolism to restore immune balance has become a promising therapeutic strategy for severe infections such as sepsis. Previously, we found that T cells in patients with ESRD exhibit a specific downregulation of CD73/ADO signaling, which was closely related to chronic inflammation, CVD and infection complications. Notably, the peripheral blood CD4 + CD73+ T-cell count in ESRD patients is significantly and inversely correlated with systemic inflammation levels, serving as an independent predictor of CVD and infection events within one year. Moreover, the decline in CD73+ T cells is more significant in severely infected individuals [16]. This suggests that impaired T-cell CD73/ADO signaling may serve as a pivotal mechanism in the immune imbalance of CKD, its progressive deterioration not only sustains chronic inflammation but also accelerates the development of infections and cardiovascular complications. In this study, our animal model further highlights that IS may play an important role in this pathological process, suggesting that treatment targeting toxins may become an important component in restoring the immune function of CKD patients.

Several limitations of this study should be acknowledged. First, we did not perform gain- or loss-of-function interventions, such as utilizing CD73 knockout/overexpression models, ADO receptor agonists/antagonists, or AhR antagonists (e.g., CH223191). Therefore, we cannot definitively establish a causal relationship at this stage. Further interventional studies are urgently needed to fully elucidate the direct causal links within the CD73/ADO and AhR pathways in this pathological process. Second, we did not verify whether clearing IS can rescue the dysfunction of the CD73/ADO signaling pathway. In this study, the chronic observation period following 5/6 nephrectomy was relatively short, which was insufficient to achieve a highly significant accumulation of endogenous IS. Future investigations employing an extended CKD modeling duration combined with adsorptive spherical carbon (an oral sorbent for IS clearance) intervention are warranted to clarify whether reducing IS load can functionally restore the T-cell CD73/ADO pathway and improve clinical outcomes. Third, the sample size was relatively small which could theoretically limit statistical power. To address this limitation transparently, we calculated and reported effect sizes and 95% CIs. Key comparisons yielded exceptionally large effect sizes for T-cell CD73 expression and serum ADO level, with 95% CIs that did not cross zero, demonstrating that our study was sufficiently powered to capture these primary pathological changes. Last but not least, we acknowledge that the lack of protein-level validation and functional ecto-nucleotidase enzymatic assays is a major limitation of this study. While our transcriptional data provide a valuable exploratory foundation, future studies utilizing flow cytometry and mass spectrometry-based purinergic flux assays are required to confirm these functional dynamics.

In conclusion, this study has revealed that IS drives chronic inflammation and exacerbates severe infections in CKD by dysregulating the T-cell CD73/ADO signaling pathway for the first time: IS can inhibit the expression of CD73 in T cells, and subsequent ADO production, thereby impairing the immunomodulatory function of this axis. On the one hand, it aggravates the basal micro-inflammatory state of CKD patients and increases the risk of cardiovascular complications. On the other hand, after infection, it further blunts host immune defenses and tissue tolerance, leading to exacerbated vascular and myocardial injury, which ultimately worsens infection severity. Our findings highlight the profound impact of PBUTs on immune function by linking IS to T-cell purinergic signaling. It is of great significance for enhancing the overall immune function of CKD patients, reducing the occurrence of major complications, and improving long-term prognosis.

## Supplementary Material

Supplementary_Material clean.docx

## Data Availability

The datasets generated and analyzed during this study are available from the corresponding author upon reasonable request.
