# Supplementary material for: Indoxyl sulfate exacerbates chronic inflammation and susceptibility to severe infection via modulating T-cell CD73/adenosine signaling in chronic kidney disease
Source: Ren Fail. 2026 Aug 5;48(1):2706213. doi: 10.1080/0886022X.2026.2706213 (PMC13446053; doi:10.1080/0886022X.2026.2706213)
Supplement: Supplementary file 1 [file IRNF_A_2706213_SM3437.docx]

**Supplementary Material**

**Indoxyl Sulfate Exacerbates Chronic Inflammation and Susceptibility to Severe Infection via Modulating T-Cell CD73/Adenosine Signaling in Uremia**

**JiLai Cao^5^, Yuxin Nie^1^, Xuesen Cao^1^, Xiaohong Chen^1^, Jin Bo^1^, Yaqiong Wang^1^, Jianzhou Zou^1^，Xiaoqiang Ding^1, 2, 3, 4^ , Bo Shen^1, 2, 3, 4^, Fangfang Xiang^1 ,2, 3, 4^**

^1^Department of Nephrology, Zhongshan Hospital, Fudan University, Shanghai 200032, China

^2^Shanghai Key Laboratory of Renal Disease and Blood Purification, Shanghai 200032, China

^3^Shanghai Medical Center of Kidney, Shanghai 200032, China.

^4^Shanghai Institute of Kidney and Dialysis, Shanghai 200032, China

^5^School of Clinical Medicine, Shanghai Medical College, Fudan University, Shanghai 200032, China

Correspondence: **Fangfang Xiang**

No.180 Fenglin Road, Xuhui District, Shanghai,China

[xiang.fangfang@zs-hosptial.sh.cn](mailto:shen.bo@zs-hosptial.sh.cn)

**Methods**

**Construction of CKD rat model**

All rats were randomly assigned to the control group, the CKD group, and the IS intervention group. The CKD models were established using the classic 5/6 nephrectomy technique for both the CKD and IS intervention groups. During the initial surgical procedure, rats were anesthetized with an intraperitoneal injection of 1% pentobarbital sodium at a dosage of 40 mg/kg. An incision was made at the left costal spine angle to expose the left kidney and to free the perirenal tissues. The renal capsule was meticulously dissected. Non-invasive forceps were employed to expose the upper and lower poles of the left kidney. Penetrating suture ligation was performed at the bases of the upper and lower poles using 4-0 silk threads, with knots tied and tightened to delineate clear ischemic lines. At the distal end of the ligation line, which was situated far from the renal hilum, the upper and lower poles were excised using ophthalmic scissors, resulting in the removal of approximately two-thirds of the left renal parenchyma. After checking for no active bleeding at the stump, the remaining renal parenchyma was returned to the abdominal cavity, and the incisions were sutured in layers. The second surgery was postponed due to the lockdown policy during the SARS-CoV-2 pandemic, a total right nephrectomy was conducted in week 12. Following the same anesthetic protocol, an incision was made at the right costal spine angle to expose the right kidney. The renal pedicle was dissected, and the renal artery and vein were ligated using 4-0 silk thread. The right kidney was then completely resected. After verifying that there was no bleeding, the muscles and skin were sutured in layers. In the sham operation control group, rats underwent only bilateral renal capsule dissection and renal dissection, without any resection of renal parenchyma. The rats in the control group received two surgical operations at the same time interval. The first operation: After anesthesia in the same way, make an incision at the left costal ridge Angle, expose the left kidney, free the perirenal tissue and separate the renal capsule, but do not perform renal pole resection. Then, suture the incision layer by layer. The second operation (one week after the first operation) : After anesthesia in the same way, make an incision at the right costal ridge Angle, expose the right kidney, free the perirenal tissue and separate the renal capsule, but do not remove the kidney. Suture the incision layer by layer. All operation steps in the sham operation group (including anesthesia duration, incision size, and free range of the kidneys) were consistent with those in the model group to eliminate the interference of surgical trauma on the experimental results. The general condition and survival of rats were closely observed after the operation. Following the successful establishment of the 5/6 Nx model, rats in the IS group received daily intraperitoneal injections of indoxyl sulfate (100μg/kg) for 24 consecutive weeks while the CKD control group was administered an equivalent volume of normal saline daily. Biological samples were collected within 2 to 6 hours after the final injection

**Construction of CLP infection model**

After intraperitoneal injection of 1% pentobarbital sodium for anesthesia in each group of rats, they were fixed on the operating table. After isoflurane anesthesia, a 1.5-cm incision was made to expose the cecum to the abdominal portion. The cecum was ligated at 70% of its total length and perforated with a single puncture midway between the ligation point and tip of the cecum using a 22-gauge needle. Once the needle was removed, extrusion of fecal matter was confirmed. The cecum was returned to its original position and the abdominal incision was closed with sutures. For fluid resuscitation, 1 mL of 0.9% normal saline, preheated to 37 °C, was subcutaneously injected into the back of the rat, which was then placed on a heating pad until complete recovery. Postoperatively, buprenorphine (0.05 mg/kg) was administered via subcutaneous injection for analgesia. In sham mice, no ligation of the cecum was performed; however, the treatment was administered identically. Antibiotics were not administered. Twenty-four hours after the CLP procedure, 5 rats of each group were euthanized and blood and tissue samples were collected. Another 5 rats in each group were used to observe the 72-hour mortality rate.

**Blood test**

Blood samples were collected via cardiac puncture at the conclusion. The blood samples were centrifuged and the sera were separated and stored at −80 °C. The concentrations of creatinine, urea nitrogen, C-reactive protein (CRP), and beta-2-microglobulin (β2-MG) were determined using enzyme-linked immunosorbent assay (ELISA) kits in accordance with the manufacturer’s instructions (Elabscience Biotechnology Co., Ltd., Wuhan, China). In addition, ELISA kits (MULTI SCIENCE, Shanghai, China) were used to quantify the levels of interleukin-1β (IL-1β), interleukin-2 (IL-2), interleukin-6 (IL-6), tumor necrosis factor-α (TNF-α), and interferon-γ (IFN-γ).

**Q****uantitative reverse transcription polymerase chain reaction**

Total RNA was extracted using TRIzol reagent (Invitrogen, Carlsbad, CA, USA), according to the manufacturer’s instructions. Reverse transcription was conducted and cDNA was prepared for amplification. The expression levels of the following genes were quantified: Rattus norvegicus ectonucleoside triphosphate diphosphohydrolase 1 (Entpd1), Rattus norvegicus5′ nucleotidase ecto (Nt5e), and Rattus norvegicus nuclear factor kappa B subunit 1 (Nfkb1). Quantification of the adenosine A2a receptor (Adora2a), adenosine A2B receptor (Adora2b), and purinergic receptor P2X 7 (P2rx7) in Rattus norvegicus was performed using an miRNA Assay Kit (Applied Biosystems, Foster City, CA, USA). Primer sequences are listed in Table S1. mRNA levels were calculated using the comparative cycle threshold (∆∆Ct) method. Relative expression levels were normalized to that of GAPDH.

**Indoxyl sulfate and p-cresyl sulfate measurement**

Briefly, 100μL of plasma was added to 500μL of an internal standard protein precipitation solution (50 ng/mL hydrochlorothiazide in methanol for IS or 50ng/mL warfarin in methanol for PCS) to precipitate the proteins. Chromatographic separation was conducted on a Venusil XBP Phenyl column (100 × 2.1 mm, 5μm; Bonna-Agela Technologies Inc., Wilmington, DE, USA) with the column temperature maintained at 40 °C. Mobile phase A comprised 2mmol/L ammonium acetate in 0.1% formic acid (v/v). Mobile phase B comprised methanol (MeOH). The mobile phase (A : B = 30 : 70) was delivered at a flow rate of 0.35 mL/min. Mass spectrometric detection was conducted on an API 3000 triple quadrupole instrument (Applied Biosystems, Toronto, ON, Canada) in multiple reaction monitoring (MRM) mode with a spray voltage of -4200V. Data were processed using the Analyst 1.4.1 software package (Applied Biosystems, Toronto, ON, Canada).

**Plasmatic quantification of nucleotides**

Briefly, the serum sample was thawed on ice, and 50 μL of the sample was mixed with 110 μL methanol (containing 100 ng/mL adenosine-13C5). The mixture was vortexed for 30 s and subsequently centrifuged at 15,000 rpm and 4 °C for 15 min. Quality control samples were obtained by isometrically pooling all prepared samples. UHPLC-MS/MS analysis was conducted using an Agilent 1290 Infinity II UHPLC system with a 6470A Triple Quadrupole mass spectrometer (Santa Clara, CA, United States). The samples were injected onto a Waters UPLC BEH Amide column (100 mm × 2.1 mm, 1.7 μm) at a flow rate of 0.2 mL/min. The mobile phase comprised two solvents, A and B, which were mixed at a 90:10 ratio. Both solvents contained 10 mM ammonium acetate (pH9; regulated by ammonium hydroxide). Chromatographic separation was conducted using a gradient elution program: the initial mobile phase composition was 90% B for the first minute, followed by a gradient elution program comprising 90% B-85% B for 4 min, 85% B-80% B for 8 min, 80%B-65%B for 7 min, 65%B-40%B for 2 min, and 40%B for 2 min. The gradient was then returned to the initial composition after 17.1 minutes, and the analysis continued for a total of 20 min. The eluted analytes were ionized using an electrospray ionization source in positive mode (ESI+). The temperature of the source drying gas and sheath gas was set to 300°C. The flow rates of the source drying and sheath gases were set to 5 and 11 L/min, respectively. The nebulizer pressure was set at 45 psi and the capillary voltage was 4000 V. The optimized MRM transition (precursor > product), fragmentor, and collision energies were determined using the MRM. Agilent MassHunter software, version B.08.00, was used for instrument control and data acquisition.
